# Implementing self-management: a mixed methods study of women’s experiences of a postpartum hypertension intervention (SNAP-HT)

**DOI:** 10.1186/s13063-020-04394-z

**Published:** 2020-06-09

**Authors:** Alexandra E. Cairns, Katherine L. Tucker, Carole Crawford, Richard J. McManus, John Powell

**Affiliations:** Nuffield Department of Primary Care Health Sciences, University of Oxford, Radcliffe Primary Care, Radcliffe Observatory Quarter, Woodstock Road, Oxford, OX2 6GG UK

**Keywords:** Hypertension, Pregnancy, Qualitative methods, Self-management, Self-monitoring

## Abstract

**Background:**

Self-management strategies are effective in a number of medical conditions; however, implementation studies have demonstrated adoption into clinical practice can be problematic. The process of implementation was explored during a pilot randomised controlled trial evaluating postpartum blood pressure self-management in women with medicated hypertensive disorders of pregnancy.

**Methods:**

A mixed methods study using semi-structured interviews with a qualitative and a scored (quantitative) component were undertaken as part of a pilot randomised controlled trial (SNAP-HT) in postnatal women with medicated gestational hypertension or pre-eclampsia. Women were randomised to usual care or blood pressure self-management. Self-management entailed daily home blood pressure monitoring and automated medication reduction via telemonitoring. Women from both groups optionally consented to participate in audio-recorded interviews at 4 weeks and 6 months postpartum. Interview questions were developed to explore the proposed benefits of self-management and the constructs of normalisation process theory. Participants provided a score (Likert scale 1–5) for each question and were encouraged to expand upon these answers through further discussion. The interviews were transcribed and analysed using the framework approach.

**Results:**

Sixty-eight women, 34 from each randomised group, completed at least one audio-recorded interview. Several overarching themes emerged from analysis of 126 interview transcripts: control; convenience; confidence, communication and knowledge; concern; constraints; and components of the intervention. In the scored component of the analysis, both groups reported feeling more in control of their condition postpartum compared to during pregnancy, more so in those self-managing at both 4 weeks and 6 months: adjusted differences 0.6 (95% confidence interval [CI] 0.2 to 1.1) and 0.7 (95% CI 0.3 to 1.2) respectively.

**Conclusions:**

Interviews and quantitative data showed that self-management enhanced women’s sense of control and improved their blood pressure-related anxiety. Prior to taking part, a few women anticipated that home monitoring might increase anxiety, but stated that it had the opposite effect. Self-management was perceived as more flexible and reactive and as enabling more targeted down-titration of medication. These data provide considerable support for blood pressure self-management postpartum and reinforce the effectiveness of the intervention used in this study.

**Trial registration:**

ClinicalTrials.gov: NCT02333240. Prospectively registered on 7 January 2015.

## Contribution to the literature:

- In the randomised trial, those self-managing had consistently lower diastolic blood pressure to 6 months postpartum [1]. This mixed methods study provides compelling adjunctive information about possible explanations for the success of self-management.
- These data showed that blood pressure self-management enhanced participants’ feelings of control, as has been demonstrated in chronic illness.
- Self-led care was perceived as more flexible, better able to detect problems and enabling more targeted medication adjustment, consistent with previous qualitative work evaluating blood pressure self-management.
- Participants reported higher levels of confidence in down-titrating medication than described previously in self-management studies of chronic hypertension involving medication up-titration.

## Background

Self-management in healthcare, and for hypertension in particular, can be beneficial to multiple stakeholders and also cost-effective [2–5]. Digital technologies are being increasingly harnessed to provide self-management solutions, with the expectation that they are cheap, widely used and acceptable [6]. However, implementation studies have shown that they are not always effectively adopted into routine clinical practice [7].

A review identified that the complex process through which innovations are implemented within organisations is often treated as a “black box”, overlooking the opportunity to understand effective implementation, a factor critical to success [8]. Linked qualitative studies have been used successfully alongside other intervention studies, including those evaluating hypertension self-management. During the TASMINH2 trial [2], semi-structured interviews, with a subset of participants undertaking self-management, showed that participants felt confident with home blood pressure monitoring (HBPM) and believed that multiple readings were more valid than those from a one-off appointment [9]. A mixed methods study (BP-Eth) sought to explore patterns of, and preferences for, different modalities of blood pressure (BP) monitoring amongst different ethnic groups. HBPM was consistently the most acceptable mode of measurement, without variation by ethnicity [10].

A mixed methods approach was used alongside a randomised controlled trial (RCT) [1] to evaluate factors influencing the successful or unsuccessful implementation of a digital intervention to support self-management of postnatal hypertension. This addressed whether patients were happy to employ self-management techniques postpartum, and what advantages or disadvantages participants perceived with this model compared to usual care.

## Methods

A pilot RCT (SNAP-HT) was conducted in women with hypertensive disorders of pregnancy (HDP) postpartum, comparing a novel self-management intervention, comprising HBPM with self-adjustment of antihypertensive medication supported by telemonitoring, with usual care [1]. Women aged ≥ 18 years with gestational hypertension or pre-eclampsia and requiring antihypertensive treatment were eligible. Exclusions were prescription of more than three antihypertensive medications, hypertension diagnosed outside pregnancy and being unable to communicate in English. During pregnancy, women from both study groups were approached face to face and given the option to provide explicit written consent for participation in audio-recorded interviews. Randomisation and the interviews took place following birth. Total population sampling was adopted: no limit was placed on the number who could be enrolled.

Semi-structured interviews provided a reproducible format for consistency, whilst allowing for further explanation and divergence. Interview questions were developed derived from the listed benefits of self-management (as stated in National Health Service [NHS] policy documents) and the constructs of normalisation process theory (NPT). NPT attempts to understand the processes by which practices are implemented, embedded and integrated into their social contexts, such that a practice becomes, and is sustained as, a routine element of everyday work [11]. There were five questions for both groups and an additional five questions for the intervention group (Additional file 1). Participants were asked to provide a score (Likert scale 1–5) for each question and were then encouraged to elaborate on their answers. These interviews were piloted before the study commenced.

Two researchers, the Chief Investigator (a doctoral student and specialty registrar doctor in Obstetrics and Gynaecology) and a research midwife, conducted the interviews. Each had training and/or experience in conducting qualitative interviews. Both researchers are female and were seeing the participants regularly during the research project. Participants were aware of the researchers’ roles in the research project. At the screening visit during pregnancy, conducted in hospital, baseline data for the first five interview questions were collected. Audio-recorded, semi-structured interviews, lasting approximately 10–15 min, were conducted during home visits at 4 weeks and 6 months after birth. The majority took place with just the interviewer and participant, but sometimes a woman’s partner was also present. Detailed field notes were not kept. The interviews were transcribed verbatim into NVivo (computer-assisted qualitative data analysis software [12]) and reviewed by the researcher for accuracy, but not returned to participants.

Transcribed interviews were coded thematically by the Chief Investigator and analysed using a framework approach [13]. This was done by review of the data from one or both time points, as available, by the participant. Analysis was both inductive and deductive: some categories were readily derived from the interview questions and informed by the prior literature review, whilst other categories and particularly the codes (subheadings) emerged from the transcriptions. The framework (Table 1) was refined during coding. After coding, framework matrices were produced for each category and code, divided by treatment group, to allow detailed data interpretation and synthesis of connections and conclusions.

**Table 1:** Coding framework

Categories	Codes	
**Access to healthcare**	Appointments	GP Midwife Practice nurse
	Continuity of care	
	Handover of care	
	Travel	
	Strategies to improve access	
**Time pressures**	New baby	
	Other children	
	Special care baby unit admission	
	Time spent on self-management	
**Communication with healthcare professionals**	Confidence in communicating with healthcare professionals	
	Listening	
	Explanations	
	Knowledge base of healthcare professionals	
**Information provision**	Information from healthcare professionals	
	Written information	
	Online information	
	Past experiences	
	Other sources of information	
**Control**	Control of BP	
	Understanding of BP	
	Responsibility sharing	
	Detection of problems	
	Adjustment of antihypertensive medication	
	Compliance with antihypertensive medication	
**Anxiety and stress**	Personal	
	Relationships	
	Impacts on lifestyle	
	Impact of BP monitoring	
**Home BP monitoring**	HBPM advantages	
	HBPM concerns	
	Sharing HBPM readings with healthcare professionals	
	White coat effect	
	Voluntary HBPM	
	Role of antenatal HBPM	
**Telemonitoring and self-management**	Ease of use	
	Suggested improvements	
	Preference for/recommendation of self-management	

Alongside this qualitative analysis, the Likert scale responses to interview questions 1–5 (asked to both groups) were analysed using a mixed effects repeated measures linear regression model. The model included the outcome with randomised group, time (as a categorical variable) and an interaction between time and randomised group as fixed effects, adjusting for recruitment site and Likert score at the screening visit, fitted as fixed effects with an unstructured covariance pattern. The differences in the adjusted mean score between the treatment groups at each time point were calculated. The correlation structure is assumed to be independent: one unique variance parameter per random effect, all covariances 0.

This dual approach aimed to bring out both articulated data, i.e. direct responses to the questions, as well as new emergent data.

The study findings are reported in line with the COnsolidated criteria for REporting Qualitative research (COREQ) (see COREQ checklist, Additional file 3) [14].

## Results

### Study participants

There were 75/91 randomised women (82%) who consented to participate in the qualitative study. Of these, 34/38 (90%) from the intervention group and 34/37 (92%) from the control group completed at least one interview. In the intervention group, 32/34 completed two interviews, and 26/34 of the control group did so as well. The baseline characteristics of the interviewees from the two groups were similar (Table 2 and Additional file 2).

**Table 2:** Baseline characteristics (*n* = 68)

Variable	Intervention (I) (***n*** = 34)	Control (C) (***n*** = 34)
**Mean age** (SD), years	32.5 (5.0)	31.9 (4.8)
**Mean BMI** (SD), kg/m^2^	28.8 (8.1)	28.5 (9.0)
**Parity** (frequency (%))		
0	22 (65%)	22 (65%)
1	8 (24%)	9 (26%)
2	2 (6%)	2 (6%)
≥ 3	2 (6%)	1 (3%)
**Ethnicity** (frequency (%))		
White (British)	29 (85%)	26 (76%)
White (other)	2 (6%)	6 (18%)
Black	1 (3%)	1 (3%)
Asian	2 6%)	1 (3%)
**IMD quintile** (frequency (%))		
1^st^	14 (41%)	17 (50%)
2^nd^	7 (21%)	6 (18%)
3^rd^	9 (26%)	6 (18%0
4^th^	4 (12%)	4 (12%)
5^th^	0 (0%)	1 (3%)
**Diagnosis** (frequency (%))		
Gestational hypertension	17 (50%)	16 (47%)
Pre-eclampsia	17 (50%)	18 (53%)
**Median gestation at diagnosis** (IQR), weeks	36.1 (34 to 37.9)	34.9 (30.9 to 36.6)
**Median gestation at delivery** (IQR), weeks	37.9 (37 to 39.3)	37.4 (36.3 to 39.1)
**Compliance with daily readings** (frequency (%))^a^		N/A
100%	9 (27%)	
75–99%	13 (39%)	
50–74%	8 (24%)	
25–49%	3 (9%)	
0–24%	0 (0%)	
**Accuracy of daily readings** (frequency (%))^b^		N/A
100%	12 (36%)	
75–99%	16 (48%)	
50–74%	3 (9%)	
25–49%	1 (3%)	
0–24%	1 (3%)	

*Abbreviations*: *BMI* body mass index, *IMD* index of multiple deprivation, *IQR* interquartile range, *N/A* not applicable, *SD* standard deviation

^a^Defined as the percentage of expected daily blood pressure readings submitted via a participant by telemonitoring; data available for 33/34 participants

^b^Defined as the percentage of submitted daily blood pressure readings which matched readings downloaded from HBPM monitor at the end of the study; data available for 33/34 participants

### Qualitative analysis

**Fig. 1:**
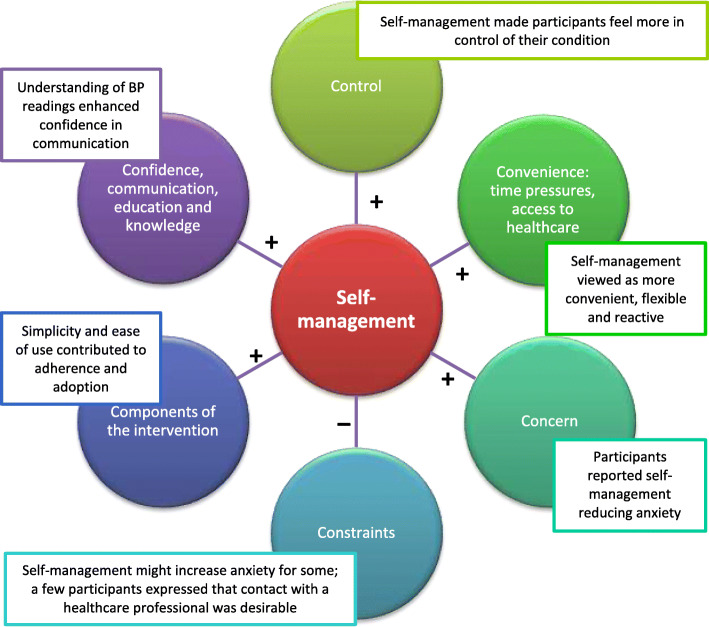
Proposed model illustrating factors positively and negatively influencing implementation and adoption of postnatal hypertension self-management

From complete coding of the 126 interview transcripts, several overarching themes emerged: control; convenience; confidence, communication and knowledge; concern; constraints; and components of the intervention (Fig. 1). Control was revealed as a major factor influencing participants’ experiences of their healthcare. Time pressures were a key concern, so ease of healthcare access was critical. There was extensive discussion surrounding communication with healthcare practitioners, patient confidence, education and knowledge, as well as evaluation of factors affecting stress and anxiety. Constraints to self-management were explored, as were participants’ positive and negative experiences of the different components of the intervention. Participants did not provide feedback on the findings.

Intriguingly, amongst the control group, 13/34 (38%) explicitly stated they had undertaken HBPM at some stage, and 21/34 (62%) unequivocally expressed a preference for HBPM.

### Control of condition

Participants frequently reflected that self-management enhanced their feelings of control: “With this study very in control, because I can check it every day, and then obviously act on that” (SM7); “every time I used to go to the doctors and come back and stuff I kind of didn’t know where I was at; it kind of felt a bit out of control, and that definitely changed post-birth” (SM25).

The views from control group participants were more inconsistent. Some were vocal that they lacked control: “I am going in to get it checked, but I don’t feel that I have much control over what happens” (UC22). Others felt that more information might have been helpful: “apart from taking the tablets, I didn’t know what my blood pressure was. It might have been nice to be able to do that” (UC15). Some participants did report feeling in control, stemming from confidence in the care provided by their general practitioner (GP): “I’m mainly making decisions alongside the GP. I just get her agreement with it” (UC1). One participant specifically valued the input from a professional: “I felt more confident that they’ve done it, rather than if I was doing it myself” (UC10).

The extent to which participants felt they shared responsibility with their healthcare team was variable. Some women who were self-managing did report sharing responsibility evenly with their GP: “it felt like he [the GP] was kind of backing it that it’s a good thing” (SM22). Others from the intervention group acknowledged that they had often assumed a greater share of the responsibility, but did not in general view this as a negative consequence of self-management: “more onus is put on me, which I think’s the right thing” (SM19). One woman from the intervention group reflected she might have liked direct contact with a healthcare professional when adjusting her medication: “normally I’d be seeing a GP and could have discussions about stopping my medication” (SM9). However, this was isolated, and this participant had some conflicting views about her management, as she also said, “My GP has not had any input into anything so I haven’t had to see him, and I was personally happy because my medication was taken away really quickly” (SM9).

Participants from the control group also reported variable levels of responsibility sharing. Some did not feel they had much input: “I don’t actually know what’s constantly going on, just the fact that it’s controlled by other people so … so you don’t really share” (UC8). Others felt they played a greater role: “I could somehow … well not really maybe influence because you can’t influence it as a patient, but still you are given the sensation you can participate in the whole thing” (UC2).

Women felt that HBPM produced a more realistic picture of their BP: “it’s more accurate and you can do it at your own sort of leisure as well” (UC34). Participants in the intervention group noted that they liked to check their BP if they developed symptoms: “if I felt … a bit funny or queasy … I could just go and check and it was kind of that reassurance” (SM25). Some women believed that they were better able to detect issues with their BP: “I wouldn’t have known it was low if I didn’t have the monitor to do it myself, and then it could have gone too low” (SM15).

Women in both groups discussed antihypertensive adjustment as a component of control. Women in the intervention group frequently referred to the speed of reduction in response to their BP readings, which in general was perceived as positive: “if I was going to the GP I would have waited two weeks and been on the full medication, and nobody really wants to be on medication if they don’t need to be” (SM31). Women felt that HBPM enabled more appropriate, and often more rapid, cessation of antihypertensive medication: “looking at how fast my medications came down, surely if I was going to see a doctor I’d still be on it without really needing to be” (SM13). One woman did express some concerns about the speed at which her medication reduced: “I think because of the monitoring that cut it down very quickly … I was almost reluctant to cut it down so quickly” (SM12), and another reported doing additional checks as the medication was decreased: “particularly when I had to reduce medication, just to check that it was holding on, so I checked at different times as well” (SM32).

Women in the control group relayed positive and negative experiences of medication adjustment: “it’s been monitored carefully and the medication reduced gradually, which I think stabilised my blood pressure” (UC16). Some women managed by their GPs did report concerns about the rate of adjustment, in both directions: “perhaps I should have come off slightly earlier, but that was my fault being busy” (UC23) and “So, I think they took, took me off it too soon” (UC7). Some participants in the control group also employed HBPM to guide adjustment: “I was also self-monitoring … I’ve called them [the GP] and said this is what my blood pressure’s doing, what, what should we do with the medication rather than waiting for an appointment” (UC9).

### Convenience: time pressures, access to healthcare and relationships with practitioners

Women explicitly referred to multiple competing time pressures and cited this as a reason why they might prefer to self-manage: “It might be good to monitor it yourself because obviously with a baby it’s a bit of a pain sometimes having to go to the doctors” (UC17). Women liked the flexibility that HBPM provided: “it’s been convenient to use it at home … I take it with me wherever I go; if I go away for a couple of days … it’s really handy” (SM20), and they felt it saved them time: “it’s time saving … if I have to go to the hospital or GP just to check my … I have to do lots of arrangements” (SM1).

Access to care was variable in both groups. Some reported that it was straightforward to get a GP appointment at their convenience: “My GPs are brilliant … they will book last minute appointments … they do cater for you” (UC29), whereas another participant recounted, “getting an appointment … it seems a sort of dark art” (UC22). Continuity emerged as an important factor influencing how women perceived the quality of care: “I try and see the same GP at the surgery and I think that helps with continuity” (UC27). Where participants achieved regular contact with their usual GP, this experience was beneficial: “I think it was once a week I used to talk to the GP … when I started going down in the medication she used to do it slowly so I didn’t have any problems” (UC12). Where continuity of care lapsed, women reported issues: “I had an interesting experience with one slightly unhelpful duty GP” (SM27).

Consistency of care was influenced by the need for handover of care, and communication between different groups of healthcare professionals. Participants reported some problems with inconsistency of message: “the community midwives completely ignored that and kept sending me back into hospital … to the point that the doctors in hospital said, ‘ … we don’t want to see you here unless your blood pressure’s above 160 and 110.’ They wrote that on my notes, and even so the community midwives kept trying to send me any time it was above 140 over 100” (SM32).

Participants reported a number of strategies employed by GP surgeries to improve access, including provision of an automated BP machine in reception, coupled with the facility to submit readings to GPs automatically: “this machine in health centre, so I always can give a receipt from these to the reception, and they usually pass it straight away to GPs” (UC3). Several participants reported effective use of telephone consultations: “my doctor was quite good in that I’d phone her and say my blood pressure’s doing this, and she’d adjust” (UC9).

Participants’ relationships with their healthcare providers affected patient care in many cases, with women frequently citing good relationships as one factor underpinning positive encounters: “I feel very confident … they’re so nice … they’re very helpful” (UC3). However, there were some reports of poor relationships negatively impacting care quality: “They’re not like my old surgery at all … I suppose I’m trying to put it off a bit going.” (UC21). Some women reported an active dislike of attending their doctors as a reason they would prefer to self-monitor: “I hate going to the doctors, and you can do it you know whenever you want at home … it’s so much easier” (SM29). Several participants relayed suffering with white coat syndrome and felt that HBPM was advantageous for them: “I found it useful just because it means I get a more accurate reading when I’m not stressed when I’m at home” (SM9). A number of participants highlighted that self-management may reduce the burden on the NHS: “Big cost saving I think for the NHS really” (SM17).

Difficulties with travel to GP surgeries were reported as a barrier to accessing healthcare. Some women were unable to drive, sometimes as a result of delivery by caesarean section, resulting in them relying on friends and family for transport: “I’ve not been able to drive, actually go [ing] to the GP and have my blood pressure checked would just be a real faff” (SM17). Other participants described difficulty in walking to appointments or sitting in waiting rooms following birth: “it was really uncomfortable to sit for half an hour, so by the time I got upstairs, my blood pressure was high” (SM33).

### Confidence, communication and knowledge

In general, participants reported high confidence levels when communicating with healthcare professionals: “I felt confident about talking to them about what was going to happen and I felt quite confident about negotiating with them” (SM4). Women strongly articulated the bearing that both adequate and inadequate understanding had upon these interactions. Positive experiences included: “I think having the element of control and knowing what my blood pressure is, um, and learning a bit more about what blood pressure actually is … it’s made me feel more confident when I’ve actually had to speak to them about it” (SM5). Women from the intervention group were explicit that specific knowledge of BP readings was helpful: “I have got control over seeing my readings, so I feel confident discussing them with them” (SM23).

The perception that they were listened to, substantially influenced participants’ experiences of interactions with healthcare professionals. Some participants reported very positive encounters: “a very good thing about her [the GP] was that she was really listening to what I was saying … we were actually collaborating” (UC2). Others’ experiences were more variable: “I got quite irate with a midwife because I felt she wasn’t listening to me” (UC13). A number of participants reflected on negative episodes: “Them listening is a different story … I feel like it was me more talking and them just sort of ticking boxes” (UC34); “I can talk to my doctor about what I think I need, but he seems to overrule me” (UC32).

Women’s views of their GPs’ understanding of HDP varied. Some felt GPs’ knowledge was good, “I think they’re very knowledgeable actually” (SM34), whilst others expressed that they did not expect their GPs to be experts, “GPs … they’re not specialists in everything so it’s hard for them to take confidence” (SM6). One participant acknowledged the difficulties GPs might face in adopting care from the hospital team: “you know the hospital controlled it all … the GP would just say, ‘Well whatever they’ve said’, rather than you know taking the step to say, ‘Right, well let’s have a look and reduce it now’” (SM7). In contrast, some women did convey concern about GPs’ and midwives’ knowledge: “they needed a bit of guidance themselves, the doctors” (UC17).

### Concern

The impact of HDP, and their management, on women’s personal anxiety and stress varied markedly. Several women reported very little intrinsic anxiety and very little effect from their condition and its management: “To be honest I didn’t really have any anxiety about it” (SM25). However, others reported this resulting in significant anxiety: “I suffer with anxiety … knowing it was high made me really concerned” (SM5). Similarly, relationships with partners, existing children, other family and friends were affected in very disparate ways. A considerable number of women reported little or no negative effects, and several in fact reported strengthening of relationships: “everyone just comes together as well so it’s not all negative; a lot of positives that comes from it as well” (UC33). Multiple women reflected that their partner had suffered considerable stress: “my poor husband’s been through the ringer … he’s more stressed” (UC30). Women also acknowledged the impact of their condition on other children, “she’s seen me get my blood pressure done, probably a bit too much for a four year old” (UC30), and other family members: “Mum always thought I was going to die” (SM15).

A concern expressed about self-monitoring is the potential to increase patients’ anxiety about their health [15]. The majority categorically stated that self-management reduced their anxiety: “It was just great having the daily check; I think that made me really calm” (SM6); “It’s reduced my anxiety and put me back in control” (SM26). One participant (SM9) held complex views of her BP management: on the one hand she reported, “it decreased it [my anxiety] as in I didn’t have to drive all these children to the doctors and wait there … Saved me some stress”. However, in stark contrast she also found having a monitor at home stressful: “I think having a doctor there to reassure me makes it easier” and “I had to have it [the monitor] sitting in my room looking at me, and I’m someone who hasn’t … just hates that”. Another participant, who reported suffering with anxiety, provided an alternative perspective: “I thought actually there might be a big negative for me. But no I don’t think it’s made me anxious … I think it’s done the opposite” (SM5).

Several women from the intervention group highlighted that self-management reduced their anxiety, relative to usual care from the GP: “going in to the doctors and things like that, yeah makes it worse for me … if they take it manually you don’t even know what it is; sometimes they don’t even tell you” (SM23); “I felt more relaxed because you let me monitor it at home. Had you made me go to the doctors, might have been a bit more worried perhaps” (SM34). Some did comment on the impacts of the standard care structure: “being told that someone’s coming out at some point today to come and like do something. So, you’re sat waiting in, nor doing something because you want to make sure that it’s OK … It was stressful to be honest” (UC34).

### Constraints

Some women reported concerns, often theoretical, about HBPM. Two acknowledged that, had they had problems, they would have wanted direct contact with a healthcare professional: “I think if I found I had very, very strong symptoms and I wanted to speak to a GP I would … ” (SM20); “if I wasn’t feeling well I would probably want, every so often still someone anyway” (SM12). Another reflected on the importance of measuring BP correctly: “when you’re doing it yourself to make, you know are you sure you’re doing it right” (UC25).

Two participants highlighted specific practical difficulties with HBPM: “actually it’s very difficult to put a blood pressure cuff on yourself when there’s nobody else at home” (SM5); “because just finding the time to sit quietly to do your blood pressure can be tricky if you’ve got no-one there to hold the baby” (SM31). A minority of women from both groups recognised that whilst they might have preferred self-management, it might not suit everyone: “like a hypochondriac it’s better not to. So I think you need to analyse the patient before” (UC12); “some people do not want to [self-monitor] maybe, or some people are not able to” (UC2); “maybe other people may like the assurance of going to a doctor” (SM25).

Participants encountered variable experiences when interacting with healthcare professionals whilst self-monitoring. Some had very positive experiences, with GPs and midwives proactively asking about readings, and being happy for patients to take ownership: “when speaking to my GP about things, again the self-checking of that it gives them reassurance that you’ve got it rather than just being reliant on the appointments” (SM19). Others, however, thought their GPs felt rather uninvolved: “the GP finding it difficult; not feeling totally informed which was partly about me bringing the paperwork with me” (SM6). Some reported healthcare professionals not trusting HBPM: “I think intrinsically they were a bit sort of dubious about whether I could do it myself and whether I would be doing it accurately” (SM27).

### Components of the intervention: participants’ experiences

Women identified specific strengths of this self-management intervention, including the speed of response: “it’s been good getting the quick responses back” (SM6). Women reported that the system was clear when instructing them how to change their medication: “sorting out my medication when the text told me to … so I found that quite good” (SM4). Some women found the reminders helpful: “I ain’t got the best memory, and they kept texting me to remind me” (SM15). Women generally found the telemonitoring services (SMS service and smartphone app) straightforward to set up and use: “they’re really easy to use; it was just the text message every day — really straightforward” (SM11). Participants also reported that the BP monitor was uncomplicated: “the machine’s straightforward” (SM21); “I think the cuff’s easy to use” (SM19).

Over the course of the trial a few isolated issues with self-management did arise. Women reported some quirks with the app, which led to them being unable to read notifications without logging into the website: “alerts come through to say there’s messages, but I can’t actually see where the messages are” (SM17). A small number of participants encountered difficulties with the pre-specified format of the SMS messages initially: “[at] the start, I didn’t notice the codes or … yeah but once that was … it was easy” (SM33).

### Quantitative analysis of pre-coded numerical responses

The first part of the semi-structured interviews was administered to both groups, and their summarised responses were compared over the trial period (Table 3). Five questions were asked; however, data relating to one question (about the impact on relationships) is not presented, as during analysis an error in how the Likert scale was used by one of the interviewers was identified (affecting only this single question).

**Table 3:** Analysis of qualitative patient experience interviews (questions 1–4,^a^ both intervention [I] and control [C] groups)

	Screening Antenatal, pre-randomisation			4 weeks			6 months		
	***I****Mean (SD)*	***C****Mean (SD)*	***Δ (I – C)****Mean (95% CI)*	***I****Mean (SD)*	***C****Mean (SD)*	***Adj. Δ (I – C)*** *Mean (95% CI)*	***I****Mean (SD)*	***C****Mean (SD)*	***Adj. Δ (I – C)***^b^ *Mean (95% CI)*
Number of responses	37	36		34	32		34	32	
*How much in control do you feel of managing your condition?*^*c*^	3.2 (1.2)	3.0 (1.4)	0.2 (– 0.4 to 0.9)	4.6 (0.7)	4.0 (1.0)	**0.6 (0.2 to 1.1)**	4.8 (0.7)	3.9 (1.1)	**0.7 (0.3 to 1.2)**
*How confident are you about taking an active part in the conversation when discussing your condition with healthcare professionals?*^*d*^	4.5 (1.0)	4.6 (0.7)	– 0.1 (– 0.5 to 0.3)	4.6 (0.6)	4.5 (1.0)	0.1 (– 0.2 to 0.4)	4.6 (0.8)	4.5 (0.8)	0.4 (0.0 to 0.7)
*How much do you feel you are sharing responsibility of your treatment with a health professional?*^*e*^	3.5 (1.4)	3.5 (1.3)	0.0 (– 0.6 to 0.6)	4.1 (1.4)	4.1 (1.1)	0.0 (– 0.7 to 0.7)	4.0 (1.3)	3.7 (1.4)	0.2 (– 0.4 to 0.9)
*How knowledgeable do you feel about your condition?*^*f*^	3.4 (1.3)	3.5 (1.0)	– 0.1 (– 0.6 to 0.5)	4.1 (0.8)	3.8 (1.0)	0.2 (– 0.2 to 0.6)	4.4 (0.7)	4.3 (0.9)	0.3 (– 0.1 to 0.7)

*Abbreviations*: *C* control, *95% CI* 95% confidence interval, *I* intervention, *SD* standard deviation

^a^Five questions were asked; however, data relating to impact on relationships are not presented here, as there was a systematic error by one of the two interviewers in how this question and answer scale was presented

^b^Adjusted difference between groups calculated using a mixed effects repeated measures regression model including outcome with randomised group, time and an interaction between time and randomised group as fixed effects, adjusting for recruitment site and question score at screening, fitted as fixed effects with an unstructured covariance pattern; adjusted differences for where the 95% CI does not cross zero are highlighted in bold

^c^Scale: 1 (do not feel in control) – 5 (very much feel in control)

^d^Scale: 1 (not confident at all) – 5 (very confident)

^e^Scale: 1 (do not feel that I am sharing responsibility) – 5 (very much feel that I am sharing responsibility)

^f^Scale: 1 (do not feel knowledgeable) – 5 (feel very knowledgeable)

Both groups felt more in control of their condition postpartum compared to during pregnancy (pre-randomisation). However, at both follow-up time points the intervention group scored this question more highly than did the control group: adjusted difference at 4 weeks 0.6 (95% confidence interval [CI] 0.2 to 1.1); adjusted difference at 6 months 0.7 (95% CI 0.3 to 1.2). For the remaining questions no significant differences were seen between groups (Table 3).

The mean scores for questions specific to the intervention group were all high (Table 4), suggesting participants felt the intervention was well suited to managing their BP, straightforward to use and that it enhanced their lifestyle. Participants were very positive that they would utilise self-management techniques in the future and would recommend them to friends and family.

**Table 4:** Analysis of qualitative patient experience interviews (questions 6–10, intervention group only)

	4 weeks Mean (SD)	6 months Mean (SD)
**Number of responses**	34	34
*The materials that you are using as part of the self-management: how well do you think they fit with managing your condition?*^*a*^	4.8 (0.4)	4.8 (0.5)
*How easy or difficult are you finding the self-management materials to operate?*^b^	4.9 (0.3)	4.9 (0.4)
*Have you seen a change in your lifestyle for better or for worse since starting self-management?*^c^	4.1 (0.9)	4.4 (0.9)
*How likely are you to recommend the self-management of gestational hypertension/pre-eclampsia to friends, family and other people?*^*d*^	4.9 (0.3)	4.9 (0.4)
*How likely are you to use self-management approaches to manage medical conditions in the future?*^e^	4.8 (0.6)	4.9 (0.4)

*SD* standard deviation

^a^Scale: 1 (do not fit with managing my condition) – 5 (fit very well with managing my condition)

^b^Scale: 1 (very difficult to operate) – 5 (very easy to operate)

^c^Scale: 1 (change in lifestyle for worse) – 5 (change in lifestyle for better)

^d^Scale: 1 (not likely) – 5 (very likely)

^e^Scale: 1 (not likely) – 5 (very likely)

## Discussion

On the whole, women reported that self-management enhanced their feelings of control and improved BP-related anxiety. This qualitative finding was supported by analysis of the coded quantitative data (Table 3). A small number reported anticipating that HBPM might increase their anxiety, but that in fact it had the opposite effect. Women thought that self-management was more flexible and reactive and that it enabled more appropriate, and often faster, down-titration of antihypertensives. As might be expected, women described multiple competing time pressures impacting their BP management. New babies, other children and work all influenced women’s ability to access healthcare and were common reasons given as to why a majority of women from both groups expressed a preference for self-management. Reinforcing this, women in the control group frequently undertook voluntary HBPM.

A few potential concerns about HBPM were raised, including the importance of ensuring correct BP measurement and the idea that self-management might not suit everyone. However, these concerns were expressed by a minority and were often theoretical in nature, rather than actual problems encountered by women self-managing.

When specifically considering the self-management intervention employed in this trial, participants commonly reflected on its simplicity, clarity and ease of use, reinforced by high mean coded scores (Table 4). Women found the reminder system helpful, with some suggesting it served as a prompt to take their medication, improving adherence. A few issues were identified, which were normally short-lived and easily resolved by the trial team. A model of factors positively and negatively influencing implementation and adoption of self-management in this trial is proposed in Fig. 1.

In contrast to the fairly consistent views of self-management, women’s experiences of doctor- and midwife-led care were more variable, perhaps reflecting the paucity of clinical guidance and evidence that currently underpins postpartum hypertension management [16, 17]. Some participants reported extremely positive experiences of shared care, open communication, thorough explanations, good continuity and easy access to healthcare. Others encountered difficulty making appointments, inconsistent messages, particularly where continuity was lacking, and felt that their views were not heard. This variation emphasises the need to establish a robust, comprehensive patient-centred model of care for postpartum hypertension management.

Themes emerging from previous qualitative work evaluating BP self-management were echoed by this study: confidence with HBPM [9] and preference for self-monitoring [10, 18, 19]. Patients have previously reported HBPM to be more straightforward, time efficient and potentially more accurate in terms of representing their true BP [19]. Participants in this cohort reported a higher level of confidence when down-titrating medication than has been reported in relation to up-titration of antihypertensive medications in poorly controlled essential hypertension [9, 20]. Those authors likened this behaviour to “clinical inertia” amongst clinicians treating borderline readings. Participant motivation to stop treatment, and not take tablets for longer than necessary, potentially alleviated this issue in the context of down- rather than up-titration.

Reclaiming and maintaining control over health have previously been identified as key consequences of self-management approaches in chronic illness, which act as positive facilitators to the implementation and sustainment of these interventions [21, 22]. A systematic review of 22 qualitative studies examining self-management strategies in diabetes identified patient empowerment as a critical emerging theme [22]. This was reiterated by a systematic review examining self-management approaches in chronic obstructive pulmonary disease, where “perceived control of worsening condition” emerged as an important positive factor in motivating patients [23]. Participants from a previous study evaluating a mobile health intervention for chronic obstructive pulmonary disease reported finding self-management reassuring, and becoming more aware of their condition as a consequence of the intervention [24]. Both themes also emerged from these data, suggesting that these may be factors that encourage adoption of self-management.

Some prior qualitative studies examining barriers and facilitators to self-management approaches in patients with essential hypertension have found substantial obstacles, including distorted views of illness course, symptoms and treatment [25, 26]. These were not identified in this study, which may reflect inherent differences between the population with essential hypertension and women with HDP, which in general have a limited time course. As hypothesised when considering this intervention, women may be more motivated in the setting of a recent pregnancy to take ownership of their health. A minority of participants did highlight the potential benefit of the regular contact with a healthcare professional encountered in usual care, previously reported as an advantage of office BP monitoring [19].

This study involved a large number of participants, evenly spread across the two groups, and the majority were interviewed at two time points. Therefore a broad range of opinions was sampled, and the potential evolution of participants’ views over the study period was accounted for. The participants were relatively diverse, covering a range of ethnicities, socioeconomic classes, parity, severity of diagnosis and gestation at delivery. During coding it became clear that saturation had been achieved: by completion of coding no new themes emerged, and the coding framework no longer required adjustment.

During analysis, no clear patterns emerged with regard to patient characteristics correlating with particular responses, suggesting that the applicability and acceptability of this self-management intervention was not confined to a particular demographic. One aspect not considered was generalisability to other languages: at present, the intervention is only available in English. A future development would be to produce versions in other languages, to expand the population to whom it would be available.

This qualitative project had a fairly narrow defined purpose, so a relatively pragmatic approach was adopted: the sample was necessarily restricted by the trial entry criteria, and the interviewers unavoidably brought their knowledge and beliefs about self-management to the interviews. Consequently, whilst informed by grounded theory, a purist inductive approach was not adhered to. The interview schedules were informed by previous research and theories, but despite these constraints, a grounded approach allowed the data to speak for itself and allowed theories to emerge. The framework approach produces a more structured output of summarised data than some other approaches to qualitative data, and it is not derived from, or aligned with, a particular philosophy or theory, but it requires reflexivity and rigour, and is a good technique for undertaking constant comparison [27].

One criticism of the study is that all participants had consented to participate in an RCT of self-management, such that they may be inherently biased towards a positive view of self-monitoring. However, the recruitment rate into the main trial was high (59% of eligible participants approached), which may have limited this bias by ensuring the study sample was more representative of the whole population. Furthermore, due to resource limitation, members of the trial team, rather than independent researchers, conducted the interviews, so participants may have felt pressured to give positive responses regarding self-management. Given their involvement in the RCT, the interviewers were inevitably positively biased towards the intervention, but the semi-structured approach helped mitigate this. By adopting total population sampling, and interviewing a large proportion of participants, it is hoped that the breadth of opinions recorded limited this bias. The small number of participants who had low compliance with, and poor accuracy of, daily HBPM were represented in the substudy sample (Table 2), which should have allowed the views of those who found self-management more challenging to be heard. Fewer women in the control group completed both interviews: it is perhaps inevitable that this group were less engaged. However, given the difference that exists in this direction, this is therefore unlikely to reflect adverse experiences with self-management which are not reflected in the qualitative data. This difference only exists for the audio-recorded interviews; the quantitative data are not affected in the same way (Table 3). The interviews were qualitatively analysed by a single reviewer, which whilst ensuring consistency of approach, may also have impacted the interpretation and conclusions.

## Conclusions

These data provide considerable support for self-management of BP postpartum, and reinforce the effectiveness of the intervention utilised in this RCT. In this cohort, self-management clearly enabled women to feel more in control of their healthcare and reduced their anxiety. Women perceived self-led care as more flexible, better able to detect newly arising problems and enabling of more accurate, targeted medication adjustment. These data may shed light on the mechanism by which self-management was effective in this study, in terms of improved BP control, as they hint at improved adherence in the intervention group and more appropriately targeted titration of medication. Most women felt comfortable measuring their BP and adjusting medication through telemonitoring, and reported that this intervention was straightforward, fit for purpose and acceptable.

## Supplementary information

**Additional file 1.** Semi-structured interview template.

**Additional file 2.** Baseline characteristics of participants.

**Additional file 3.** COREQ (COnsolidated criteria for REporting Qualitative research) checklist.

## Data Availability

Data sharing requests should be directed to information.guardian@phc.ox.ac.uk.

## Abbreviations

BMI: Body mass index
BP: Blood pressure
CI: Confidence interval
GP: General practitioner
HBPM: Home blood pressure monitoring
HDP: Hypertensive disorders of pregnancy
IMD: Index of multiple deprivation
IQR: Interquartile range
NPT: Normalisation process theory
RCT: Randomised controlled trial
SD: Standard deviation
