# Supplementary material for: Implementing self-management: a mixed methods study of women’s experiences of a postpartum hypertension intervention (SNAP-HT)
Source: Trials. 2020 Jun 9;21:508. doi: 10.1186/s13063-020-04394-z (PMC7282057; doi:10.1186/s13063-020-04394-z)
Supplement: Supplementary file 1 — Additional file 1. Semi-structured interview template. [file 13063_2020_4394_MOESM1_ESM.docx]

**Additional File 1: Semi-structured interview template**

| Researcher initials/ Date of interview | *e.g. AC / 27-Aug-2014* | | |  | | Participant’s Study ID | SNAP- - |
| --- | --- | --- | --- | --- | --- | --- | --- |
|  |  | | | | | | |
| Record group | *Intervention / Control* | | | |  | Visit number (1/4/7) |  |
|  |  | | | | | | |
| **We are going to ask you some questions that relate to the raised blood pressure you have developed during your pregnancy.** | | | | | | | |
| **Part 1** | **For all participants (including those in the control group) at visits 1, 4 and 7** | | | | | | |
|  | | | | | | | |
| **On a scale of 1 to 5 (1 being the least and 5 being the most) please indicate how participant rated the following questions:** | | | | | | | |
| 1.  How much in control do you feel of managing your condition? | 1. Do not feel in control | | Explain why: | | | | |
|  | 2. | |  |  |  |  |  |
|  | 3. | |  |  |  |  |  |
|  | 4. | |  |  |  |  |  |
|  | 5. Very much feel in control | |  |  |  |  |  |
|  |  | |  |  |  |  |  |
| 2.  How confident are you about taking an *active* part in the conversation when discussing your condition with health care professionals? | 1. Not confident at all | | Explain why: | | | | |
|  | 2. | |  |  |  |  |  |
|  | 3. | |  |  |  |  |  |
|  | 4. | |  |  |  |  |  |
|  | 5. Very confident | |  |  |  |  |  |
|  |  | |  |  |  |  |  |
| 3.  How much do you feel you are sharing responsibility of your treatment with a health professional? | 1. Do not feel that I am sharing responsibility | | Explain why: | | | | |
|  | 2. | |  |  |  |  |  |
|  | 3. | |  |  |  |  |  |
|  | 4. | |  |  |  |  |  |
|  | 5. Very much feel that I am sharing responsibility | |  |  |  |  |  |
|  | *Continued overleaf* | |  |  |  |  |  |
| 4.  How knowledgeable do you feel about your condition? | 1. Do not feel knowledgeable | | Explain why: | | | | |
|  | 2. | |  |  |  |  |  |
|  | 3. | |  |  |  |  |  |
|  | 4. | |  |  |  |  |  |
|  | 5. Feel very knowledgeable | |  |  |  |  |  |
|  |  | |  |  |  |  |  |
| 5.  To what extent has your condition impacted on your relationships with family and friends? | 1. Not able to maintain good relationships | | Explain why: | | | | |
|  | 2. | |  |  |  |  |  |
|  | 3. | |  |  |  |  |  |
|  | 4. | |  |  |  |  |  |
|  | 5. Very much able to maintain good relationships | |  |  |  |  |  |
|  |  | |  |  |  |  |  |
|  | | | | | | | |
| ***Continue to part two at visit 4 and 7 if participant is part of intervention group*** | | | | | | | |
|  | | | | | | | |
| **Part 2** | **For the intervention group only at visits 4 and 7** | | | | | | |
| 6.  The materials that you are using as part of the self-management: how well do you think they fit with managing your condition? | 1. Do not fit with managing my condition | Explain why: | | | | | |
|  | 2. |  |  |  |  |  |  |
|  | 3. |  |  |  |  |  |  |
|  | 4. |  |  |  |  |  |  |
|  | 5. Fit very well with managing my condition |  |  |  |  |  |  |
|  |  |  |  |  |  |  |  |
| 7.  How easy or difficult are you finding the self-management materials to operate? | 1. Very difficult to operate | Explain why: | | | | | |
|  | 2. |  |  |  |  |  |  |
|  | 3. |  |  |  |  |  |  |
|  | 4. |  |  |  |  |  |  |
|  | 5. Very easy to operate |  |  |  |  |  |  |
|  | *Continued overleaf* |  |  |  |  |  |  |
|  | | | | | | | |
| 8.  Have you seen a change in your lifestyle for better or for worse since starting self-management? | 1. Change in lifestyle for worse | Explain why: | | | | | |
|  | 2. |  |  |  |  |  |  |
|  | 3. |  |  |  |  |  |  |
|  | 4. |  |  |  |  |  |  |
|  | 5. Change in lifestyle for better |  |  |  |  |  |  |
|  |  |  |  |  |  |  |  |
| 9.  How likely are you to recommend the self-management of gestational hypertension / pre-eclampsia to friends, family and other people? | 1. Not likely | Explain why: | | | | | |
|  | 2. |  |  |  |  |  |  |
|  | 3. |  |  |  |  |  |  |
|  | 4. |  |  |  |  |  |  |
|  | 5. Very likely |  |  |  |  |  |  |
|  |  |  |  |  |  |  |  |
| 10.  How likely are you to use self-management approaches to manage medical conditions in the future? | 1. Not likely | Explain why: | | | | | |
|  | 2. |  |  |  |  |  |  |
|  | 3. |  |  |  |  |  |  |
|  | 4. |  |  |  |  |  |  |
|  | 5. Very likely |  |  |  |  |  |  |
|  |  |  |  |  |  |  |  |
| Additional notes / researcher reflections: | | | | | | | |
