# Supplementary material for: Implementing self-management: a mixed methods study of women’s experiences of a postpartum hypertension intervention (SNAP-HT)
Source: Trials. 2020 Jun 9;21:508. doi: 10.1186/s13063-020-04394-z (PMC7282057; doi:10.1186/s13063-020-04394-z)
Supplement: Supplementary file 2 — Additional file 2. Baseline characteristics of participants. [file 13063_2020_4394_MOESM2_ESM.docx]

# Additional File 2: Baseline characteristics of participants

Baseline characteristics of interviewees from self-management group (n=34)

| **Interviewee ID** | **Study site** | **Withdrawn** | **Age**/y | **Ethnicity** | **IMD quintile** | **Parity** | **Diagnosis** | **Gestation at delivery /**weeks | **% compliance** (daily HBPM) | **% accuracy** (daily HBPM) |
| --- | --- | --- | --- | --- | --- | --- | --- | --- | --- | --- |
| SM1 | Oxford | No | 37 | Indian | 4 | 2 | PET | 33.9 | 66.7% | 94.4% |
| SM2 | Oxford | No | 31 | White British | 1 | 1 | PET | 36.6 | 64.7% | 100.0% |
| SM3 | Oxford | No | 34 | White British | 3 | 0 | PET | 32.3 | 63.6% | 90.9% |
| SM4 | Oxford | No | 34 | White British | 3 | 0 | PET | 38.3 | 100.0% | 100.0% |
| SM5 | Oxford | No | 32 | White British | 2 | 0 | GH | 38.9 | 85.7% | 96.9% |
| SM6 | Oxford | No | 36 | White British | 1 | 1 | PET | 37.0 | 80.0% | 87.5% |
| SM7 | Oxford | No | 31 | Pakistani | 1 | 1 | PET | 36.3 | 61.1% | 95.5% |
| SM8 | Oxford | No | 36 | White British | 2 | 0 | PET | 38.7 | 80.7% | 94.1% |
| SM9 | Oxford | No | 34 | White British | 2 | 2 | GH | 39.1 | 44.4% | 50.0% |
| SM10 | Oxford | No | 40 | White British | 2 | 0 | GH | 37.0 | 50.0% | 100.0% |
| SM11 | Oxford | No | 34 | White British | 3 | 0 | PET | 38.0 | 90.0% | 100.0% |
| SM12 | Oxford | No | 43 | White other | 1 | 1 | GH | 37.1 | 85.7% | 57.1% |
| SM13 | Oxford | No | 28 | White British | 1 | 0 | PET | 38.0 | 100.0% | 100.0% |
| SM14 | Oxford | No | 36 | White British | 3 | 3 | GH | 39.3 | 95.2% | 100.0% |
| SM15 | Oxford | No | 26 | White British | 1 | 0 | PET | 36.4 | 92.3% | 92.9% |
| SM16 | Oxford | No | 23 | White British | 1 | 0 | PET | 37.7 | 60.6% | 28.6% |
| SM17 | Oxford | No | 31 | White British | 3 | 0 | PET | 37.9 | 100.0% | 100.0% |
| SM18 | Banbury | Yes | 29 | White British | 3 | 0 | GH | 39.1 |  |  |
| SM19 | Reading | No | 35 | White British | 1 | 0 | GH | 40.6 | 100.0% | 96.7% |
| SM20 | Reading | No | 29 | White British | 2 | 0 | GH | 37.1 | 100.0% | 100.0% |
| SM21 | Reading | No | 29 | White British | 1 | 1 | GH | 38.1 | 38.9% | 75.0% |
| SM22 | Reading | No | 26 | White British | 3 | 0 | PET | 36.0 | 43.2% | 20.0% |
| SM23 | Reading | No | 28 | White British | 2 | 0 | GH | 37.4 | 98.4% | 87.3% |
| SM24 | Reading | No | 34 | White British | 1 | 0 | GH | 40.9 | 93.0% | 95.2% |
| SM25 | Reading | No | 28 | White British | 1 | 0 | GH | 40.0 | 73.9% | 94.1% |
| SM26 | Reading | No | 39 | White British | 1 | 1 | GH | 39.9 | 90.9% | 100.0% |
| SM27 | Reading | No | 31 | White British | 3 | 1 | PET | 39.3 | 100.0% | 100.0% |
| SM28 | Reading | No | 25 | White British | 1 | 0 | GH | 40.7 | 85.7% | 100.0% |
| SM29 | Reading | No | 39 | White British | 1 | 0 | PET | 30.0 | 98.5% | 100.0% |
| SM30 | Northampton | No | 41 | African | 3 | 3 | PET | 32.1 | 74.1% | 63.6% |
| SM31 | Northampton | No | 36 | White British | 4 | 0 | GH | 39.7 | 100.0% | 90.9% |
| SM32 | Aylesbury | No | 29 | White other | 2 | 1 | GH | 37.6 | 79.1% | 97.3% |
| SM33 | Aylesbury | No | 37 | White British | 4 | 0 | PET | 37.4 | 100.0% | 90.0% |
| SM34 | Aylesbury | No | 25 | White British | 4 | 0 | GH | 39.4 | 100.0% | 98.4% |

Abbreviations: GH = gestational hypertension; HBPM = home blood pressure monitoring; IMD = index of multiple deprivation; PET = pre-eclampsia

Baseline characteristics of interviewees from usual care group (n=34)

| **Interviewee ID** | **Study site** | **Withdrawn** | **Age** /y | **Ethnicity** | **IMD quintile** | **Parity** | **Diagnosis** | **Gestation at delivery** /weeks |
| --- | --- | --- | --- | --- | --- | --- | --- | --- |
| UC1 | Oxford | No | 32 | White British | 1 | 1 | PET | 36.6 |
| UC2 | Oxford | No | 36 | White other | 4 | 0 | GH | 40.0 |
| UC3 | Oxford | No | 34 | White other | 5 | 1 | PET | 39.0 |
| UC4 | Oxford | Yes | 25 | White British | 1 | 0 | PET | 29.0 |
| UC5 | Oxford | No | 26 | White other | 2 | 0 | GH | 37.1 |
| UC6 | Oxford | Yes | 34 | White British | 3 | 0 | PET | 36.6 |
| UC7 | Oxford | No | 23 | White British | 3 | 0 | PET | 37.9 |
| UC8 | Oxford | No | 32 | White British | 1 | 0 | GH | 37.1 |
| UC9 | Oxford | No | 39 | White British | 2 | 0 | GH | 36.3 |
| UC10 | Oxford | No | 36 | White other | 1 | 0 | PET | 37.4 |
| UC11 | Oxford | No | 37 | White British | 1 | 0 | GH | 40.3 |
| UC12 | Oxford | No | 31 | White other | 1 | 0 | PET | 27.9 |
| UC13 | Oxford | No | 29 | White British | 3 | 1 | PET | 37.9 |
| UC14 | Oxford | No | 35 | White British | 3 | 1 | GH | 40.3 |
| UC15 | Oxford | No | 34 | White British | 1 | 1 | PET | 38.6 |
| UC16 | Oxford | No | 30 | African | 3 | 3 | PET | 31.6 |
| UC17 | Oxford | No | 31 | White British | 1 | 0 | GH | 39.9 |
| UC18 | Banbury | No | 30 | White British | 1 | 0 | GH | 39.6 |
| UC19 | Reading | No | 31 | White British | 2 | 1 | PET | 36.4 |
| UC20 | Reading | No | 28 | White British | 1 | 1 | PET | 39.3 |
| UC21 | Reading | No | 31 | White British | 1 | 0 | GH | 41.1 |
| UC22 | Reading | No | 34 | White British | 1 | 0 | PET | 35.3 |
| UC23 | Reading | No | 31 | White British | 4 | 0 | GH | 37.4 |
| UC24 | Reading | No | 40 | White British | 1 | 0 | GH | 36.3 |
| UC25 | Reading | No | 41 | White British | 1 | 1 | GH | 39.4 |
| UC26 | Reading | No | 39 | Asian other | 2 | 0 | GH | 39.1 |
| UC27 | Reading | No | 38 | White British | 1 | 2 | GH | 38.3 |
| UC28 | Reading | No | 29 | White British | 4 | 0 | PET | 31.6 |
| UC29 | Reading | No | 29 | White British | 2 | 2 | GH | 38.4 |
| UC30 | Reading | No | 33 | White other | 2 | 1 | PET | 33.0 |
| UC31 | Reading | No | 31 | White British | 1 | 0 | GH | 39.0 |
| UC32 | Northampton | No | 28 | White British | 4 | 0 | PET | 35.0 |
| UC33 | Northampton | No | 23 | White British | 3 | 0 | PET | 32.6 |
| UC34 | Northampton | No | 23 | White British | 1 | 0 | PET | 37.4 |

Abbreviations: GH = gestational hypertension; IMD = index of multiple deprivation; PET = pre-eclampsia
